# The effect of size-segregated ambient particulate matter on Th1/Th2-like immune responses in mice

**DOI:** 10.1371/journal.pone.0173158

**Published:** 2017-02-28

**Authors:** Kuo-Liang Huang, Szu-Yuan Liu, Charles C. K. Chou, Yi-Hsin Lee, Tsun-Jen Cheng

**Affiliations:** 1 Division of Pulmonary Medicine, Taipei Tzu Chi Hospital, Buddhist Tzu Chi Medical Foundation, New Taipei City, Taiwan; 2 Institute of Occupational Medicine and Industrial Hygiene, College of Public Health, National Taiwan University, Taipei, Taiwan; 3 Research Center for Environmental Change, Academia Sinica, Taipei, Taiwan; 4 Department of Pathology, Taipei Tzu Chi Hospital, Buddhist Tzu Chi Medical Foundation, New Taipei City, Taiwan; Centre National de la Recherche Scientifique, FRANCE

## Abstract

**Background:**

Particulate matter (PM) has been associated with increased pulmonary and cardiovascular mortality and morbidity. Additionally, PM is known to exacerbate asthma. However, whether ambient PM exposure contributes to the onset of asthma, especially in non-atopic children and adults, is less conclusive. The current study aimed to evaluate the effects of size-fractioned PM on lung immune responses in healthy BALB/c mice.

**Methods and principal findings:**

We collected PM_10_, PM_2.5_, PM_1_ and PM_0.1_ samples from October 2012 to August 2013 in the Taipei Basin. These PM samples were representative of urban traffic pollution. The samples were extracted and sonicated in phosphate-buffered saline (PBS). Female BALB/c mice were exposed to the samples via intratracheal instillation at three different doses: 1.75 mg/kg (35 μg/per mouse), 5 mg/kg (100 μg/per mouse), and 12.5 mg/kg (250 μg/per mouse). The mice were exposed on days 0 and 7, and PBS alone was used as a control. Following the exposures, the expression profiles of inflammatory cells and cytokines in bronchoalveolar lavage fluid (BALF) were assessed. Exposure to PM_10_ resulted in inflammatory responses, including the recruitment of neutrophils and the induction of T helper 1 (Th1) cell-related cytokine release, such as TNF-α and IFN-γ. Furthermore, an allergic immune response, including the recruitment of eosinophils and the up-regulation of T helper 2 (Th2) cell-related cytokine release, such as IL-5 and IL-13, was also observed in the BALF of mice exposed to PM_10_.

**Conclusions:**

Our study showed that exposure to PM alone caused mixed Th1/Th2 inflammatory responses in healthy mice. These findings support the hypothesis that PM may contribute to the onset of asthma.

## Introduction

Particulate matter (PM) is the major component of air pollution, which includes emissions from both anthropogenic and natural sources. Based on its aerodynamic diameter, PM is crudely categorized as coarse PM, which has an aerodynamic diameter of 2.5–10 μm; fine PM, which has an aerodynamic diameter of <2.5 μm; and ultrafine PM, which has an aerodynamic diameter of <0.1 μm. Fine PM is small enough to penetrate alveoli and terminal bronchioles, while coarse PM is primarily deposited in large conducting airways [1]. PM_10_ (aerodynamic diameter <10 μm) includes coarse, fine and ultrafine PM. The sizes and components of the PM depend on its source. For example, ultrafine PM is predominantly derived from primary combustion emissions or gas-to-particle conversion processes and consists of sulfates, nitrates, organic carbon (OC) and elemental carbon (EC). Coarse PM contributes the major proportion of total particle mass in smoke, soil from roads and construction sites. It can also contain sea salts, molds, dust mites, pollen and spores [2]. As industrialization and urbanization have increased, diesel exhaust particles (DEPs) have become a major source of ambient PM in modern cities. DEPs are composed of an elemental carbon core to which hundreds of chemicals and transition metals are attached [3]. Evidence suggests that PM is associated with increased pulmonary and cardiovascular morbidity and mortality [4–6].

Asthma is a chronic inflammatory disease of the airways and the most common chronic disease among children [7]. The World Health Organization (WHO) estimates that 1–2% of total health care expenditures in developed countries are associated with treating asthma [8]. The prevalence of asthma is also increasing in many countries, including Taiwan [9]. A study conducted by the International Study of Asthma and Allergies in Childhood (ISAAC) reported that the prevalence of asthma symptoms shows a high degree of global variation, even within genetically similar groups [10]. This suggests that environmental factors, such as allergens, viruses and PM, may be responsible for some of this variation.

Epidemiological reports have shown that PM can exacerbate asthma [11–13]. However, whether ambient PM exposure contributes to the onset of asthma, especially in non-atopic children and adults, is less conclusive. An epidemiological study showed that exposure to PM may adversely affect lung function development between the ages of 10 and 18 years [14]. Exposure to PM_10_ in utero and during the first year of life has also increased the risk of asthma development [15]. A cross-sectional epidemiological study conducted in Germany suggested that the composition of PM in the study area may have contributed to the high asthma prevalence there [16]. In a heavily industrialized province of China, investigators found that PM_10_ was related to asthma prevalence among children without an allergic predisposition [17]. In a prospective study of the World Trade Center (WTC) disaster, those who were exposed to WTC dust showed bronchial hyperactivity, persistent cough and increased risk of asthma [18]. A human challenge study found that inhaled coarse fraction PM may activate monocytic cells and potentially result in an allergic response in airways [19]. These studies suggest that long-term time-weighted averages of or short-term peak exposure to PM may be responsible for new-onset asthma.

Murine asthma models exhibit important features of allergic asthma, including the development of airway hyper-responsiveness, increased serum IgE levels, increased eosinophil counts and augmented T helper 2 cell (Th2) cytokine expression (e.g., IL-4, IL-5, and IL-13). Therefore, these models have been widely used to study the impact of environmental factors on asthma development [20]. For example, an ovalbumin (OVA)-induced mouse model of allergic airway disease was used to validate findings from an epidemiological study demonstrating that PM_2.5_ composition may influence the severity of allergic airway inflammation both during sensitization and the challenge phase [21]. Li et al. [22] found that the inhalation of ambient ultrafine particles enhanced the secondary immune response to OVA in sensitized mice, suggesting that exposure to vehicle exhaust can exacerbate allergic inflammation in asthmatic subjects. Most previous *in vivo* studies have addressed the adjuvant effects of PM on allergic airway inflammation [21–24], whereas few have investigated the pure PM effect. We hypothesized that repeated exposure to ambient PM without additional OVA treatment would promote allergic airway inflammation.

In this study, we used a murine intratracheal sensitization model and different doses of size-fractionated PM collected from air samplers in the Taipei Basin to determine how PM contributes to the development of Th2 immune responses in healthy mice.

## Materials and methods

### PM collection and preparation

PM samples were collected at the Atmospheric Science Building of the National Taiwan University (NTU) (25.0°N, 121.5°E) in Taipei, Taiwan from October 2012 to August 2013. The NTU is located in the downtown area of the Taipei Basin and therefore provides representative data for the PM that exists in a subtropical urban area. PQ200 Air Samplers (Model PQ200, BGI Inc., MA, USA) were used to collect PM_10_, PM_2.5_, and PM_1_ (aerodynamic diameter <1.0 μm) samples at a flow rate of 16.67 L/min. MOUDIs (Model 110, MSP Corp., MN, USA) were used to collect PM_0.1_ samples at a flow rate of 30.0 L/min in the sampling campaigns. Two aerosol samples were collected for each condition on polytetrafluoroethylene (PTFE) and quartz fiber filters. The sampling period for each condition was 22 h: from 13:00 LST to 11:00 LST the next day. The PTFE samples were used for animal studies, and the quartz filter samples were used for composition analysis.

#### Chemical characterization of PM samples

All quartz filters were pre-heated at 900°C for 3 h and then stored in aluminum foil before sampling. After sampling, the filters were immediately placed in a cooler packed with ice and transported to the laboratory within 30 min. The samples were then stored in a freezer at -18°C until OC/EC analysis, which occurred within 3 days. All loaded filters for PM_10_, PM_2.5_, PM_1_ and PM_0.1_ were analyzed for OC and EC content using a DRI Model 2001 Thermal/Optical Carbon Analyzer (Atmoslytic Inc., Calabasas, CA, USA). Additionally, the following ion species were analyzed using an ion chromatograph (IC, Model DX-120, Dionex Corp, Sunnyvale, CA): Na^+^, K^+^, Mg^2+^, Ca^2+-^, Cl^-^, NH_4_^+^, NO_3_^-^, PO_4_^3-^ and SO_4_^2-^. Endotoxin levels in PBS-eluted PM samples collected on PTFE filters were determined using a Limulus amebocyte lysate assay according to the manufacturer’s instructions (QCL-1000, CAPE COD, USA).

#### Preparation of PM samples for animal studies

Pre-weighed PTFE filters stored at −18°C were acclimated to room temperature (16–20°C, 30–40% relative humidity) prior to the elution of PM. The filters were placed into labeled 15 ml conical vials. A 1 ml aliquot of phosphate-buffered saline (PBS) was added to each conical vial. Elution was performed in water with water bath sonication for 30 min. The filters were weighed before and after elution using an analytical balance. The eluted contents were pooled into one vial and then diluted in PBS to final concentrations of 35 μg/100 μL, 100 μg/100 μL, and 250 μg/100 μL. All eluted PM samples were stored as aliquots at −80°C until 30 min prior to experimentation.

### Animals

Eight-week-old female BALB/c mice (~20 g each) were obtained from the National Laboratory Animal Center (Taipei, Taiwan) and maintained under a 12 h light/dark cycle at a constant temperature of 22 ± 2°C and 55 ± 10% relative humidity throughout the study. The animals were housed in plastic cages and had access to LabDiet 5001 food (PMI Nutrition International, Brentwood, MO, USA) and water *ad libitum*. The animals were acclimated for one week prior to the initiation of the study. The care and handling of the animals were in accordance with the Guidelines for the Care and Use of Laboratory Animals at National Taiwan University. This study was approved by the Institutional Animal Care and Use Committee of NTU (Permit Number: 20110508).

### Experimental design

**Fig 1:**
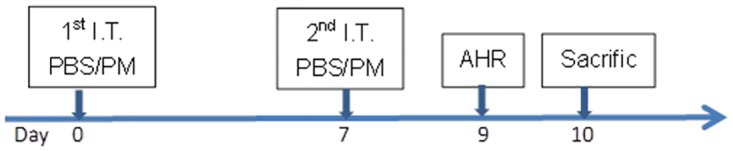
Overview of the experimental design used to expose mice to PM. Mice (10–15 animals per group) were divided into the following five groups: PBS, PM_0.1_, PM_1_, PM_2.5_, and PM_10_. Each group was further divided according to dose: either two or three doses of 1.75 mg/kg (35 μg/per mouse), 5 mg/kg (100 μg/per mouse) or 12.5 mg/kg (250 μg/per mouse) were administered. On days 0 and 7, the mice were exposed to 100 μL of the appropriate solution by intratracheal instillation under light anesthesia. The animals were sacrificed 3 days after the second treatment, and samples of blood, BALF and lung were collected.

The mice (10–15 animals per group) were divided into the following five groups: PBS, PM_0.1_, PM_1_, PM_2.5_, and PM_10_. Each group was further divided based on treatment dose; three different doses were evaluated: 1.75 mg/kg (low dose, 35 μg/per mouse), 5 mg/kg (medium dose, 100 μg/per mouse) and 12.5 mg/kg (high dose, 250 μg/per mouse). We referred to previous PM studies to determine the exposure doses [25,26]. Due to the limited amount of PM collected, a high-dose group was not established for PM_0.1_. On days 0 and 7, the mice were exposed to 100 μL of the appropriate solution by intratracheal instillation (IT) under light anesthesia (Sevoflurane; Abbott Laboratories, UK). Briefly, each mouse was placed in a supine position at an elevation of 45–60 degrees, and its mouth was opened by hanging its incisors on a small wire. The solution was then instilled into the trachea via gavage needle under a laryngoscope. After the instillation, the mouse was closely observed until it fully recovered to ensure that suffocation was avoided. The animals were sacrificed 3 days after the second treatment. Blood, bronchoalveolar lavage fluid (BALF) and lung samples were collected (Fig 1). To achieve the minimum amount of experimental animals, we measured eosinophils and Th2 cytokines in the lung lavage fluid at same time because they play major roles in allergic airway inflammation. The protocol was modified from our previous study, which showed that the Th2 cytokine levels increased at 24 h, and eosinophilia peaked at 7 days in the BALF after ZnO nanoparticle exposure [27]. We conducted these independent experiments at intervals of 1 month apart (20–25 mice per independent experiment).

#### Determination of total serum IgE and IL-6 levels

We obtained blood samples from the facial veins of the mice. Total serum IgE was measured using a Mouse IgE ELISA Kit (BD Biosciences, San Jose, CA, USA), and serum IL-6 concentrations were measured using mouse-specific IL-6 ELISAs (BD Biosciences, San Jose, CA, USA) according to the manufacturer’s instructions.

#### Animal sacrifice, sample collection and analysis

The animals were sacrificed using an overdose of sodium pentobarbital (0.1 ml at 200 mg/ml) administered via intraperitoneal injection. Samples of BALF and left lung tissue were collected. Then, BALF was prepared for the subsequent analysis. The total protein concentration was determined using a total protein assay kit (Bio-Rad Hercules, CA, USA). Total cell counts were determined from fresh fluid specimens using a hemocytometer after trypan blue staining. Differential cell counts were measured on cytological slides with Liu’s stain (Tonyar Biotech, Taiwan). Inflammatory cytokines in the BALF were measured using a Cytometric Bead Array Mouse Enhanced Sensitivity Flex Set system (BD BioSciences, San Diego, CA, USA). The detailed procedures were previously described by us [27].

#### Lung histology

The left lung lobes of the mice were excised, fixed with 10% neutral phosphate-buffered formalin, embedded in paraffin, sectioned at 5-μm thickness and stained with hematoxylin and eosin (H&E). The histological samples were examined in a blinded manner under a light microscope by an experienced histopathologist.

### Statistical analysis

All animal study results are expressed as the mean ± SEM. Differences between groups were evaluated by analysis of variance (ANOVA), and Turkey’s post hoc *t*-test for multiple comparisons was used to distinguish between pairs of groups. Relationships between PM constituents and inflammatory variables of all tested doses were analyzed using Spearman’s rank correlation test. All statistical analyses in this study were performed with SAS software (SAS Institute, Cary. NC, USA), version 9.3. The level of statistical significance was set at *p*<0.05.

## Results

### Characterization of PM

The chemical mass constituents found in the size-segregated ambient PM samples collected between the 2012 winter and 2013 summer seasons are shown in Table 1. Sulfate was identified as the major constituent in the samples, accounting for 25.65% of the PM_10_ mass, followed by OC, NH_4_^+^ and NO_3_^-^. The PM_2.5_ and PM_1_ samples showed similar mass proportions of major constituents to those found in PM_10_. OC and EC contents were highly enriched in the PM_0.1_ sample. The characteristics and compositions of PM samples collected at a nearby central air monitor station in Taipei Basin have been previously reported [28]. In general, soil dust was the major source of PM_10_, followed by vehicle emissions, secondary aerosols, sea salts and industrial emissions. For PM_2.5_, vehicle emissions were the major aerosols, followed by industrial emissions and secondary aerosols. Secondary aerosols and vehicle emissions were the major sources of PM_0.1_. The highest endotoxin levels were detected in the PM_10_ samples.

**Table 1:** Chemical mass constituents in size-segregated ambient PM collected between the 2012 winter and 2013 summer seasons.

	PM0.1	PM1	PM2.5	PM10
Collected mass (mg)	1.87	19.48	26.17	40.88
Ionic components (μg/mg)				
Na^+^	3.44	3.84	13.72	31.73
K^+^	6.92	13.19	12.67	10.78
Mg^2+^	1.33	0.59	2.08	4.92
Ca^2+^	7.87	1.49	3.34	14.68
Cl^-^	5.12	2.78	9.77	18.90
NH_4_^+^	11.31	125.34	119.93	75.76
NO_3_^-^	17.46	49.37	87.91	96.19
PO_4_^3-^	7.47	1.37	1.68	1.45
SO_4_^2-^	45.28	349.90	318.69	240.19
Carbon species				
OC (μg/mg)	314.02	120.51	166.74	126.14
EC (μg/mg)	47.53	53.05	46.31	41.60
Endotoxin (EU/ml)	62.44	53.66	55.05	122.27

### Serum total IgE, serum IL-6 and total protein in BALF

Total serum IgE and IL-6 levels did not significantly increase following the intratracheal instillation of different doses or different sizes of PM. BALF total protein content did not significantly differ between any of the treatment groups (Table 2).

**Table 2:** Serum total IgE, serum IL-6 and total protein in BALF.

	N	Serum IgE (ng/ml)	Serum IL-6 (pg/ml)	Total Protein (mg/ml)
PBS	5	676.4 (±220.8)	5.41 (±0.64)	0.28 (±0.02)
Low dose PM				
PM_0.1_ 35 μg	5	968.0 (±311.9)	6.68 (±1.16)	0.30 (±0.03)
PM_1.0_ 35 μg	5	575.1 (±165.7)	6.43 (±0.65)	0.31 (±0.04)
PM_2.5_ 35 μg	5	424.7 (±113.1)	6.41 (±0.91)	0.27 (±0.03)
PM_10_ 35 μg	5	513.9 (±164.5)	6.09 (±0.43)	0.23 (±0.02)
Medium dose PM				
PM_0.1_ 100 μg	5	630.6 (±98.7)	6.58 (±1.04)	0.31 (±0.03)
PM_1.0_ 100 μg	5	419.0 (±64.5)	6.64 (±1.29)	0.35 (±0.05)
PM_2.5_ 100 μg	5	445.9 (±60.2)	5.97 (±0.95)	0.33 (±0.04)
PM_10_ 100 μg	5	547.4 (±118.5)	5.42 (±1.23)	0.29 (±0.05)
High dose PM				
PM_1.0_ 250 μg	5	440.7 (±95.5)	8.27 (±1.46)	0.22 (±0.01)
PM_2.5_ 250 μg	5	465.9 (±87.5)	6.83 (±0.98)	0.34 (±0.03)
PM_10_ 250 μg	5	406.9 (±64.1)	5.64 (±0.94)	0.34 (±0.02)

The mice were exposed to 100 μL solutions by intratracheal instillation on days 0 and 7 as described in the Materials and Methods section.

The data are presented as the mean ± SEM.

### Inflammatory cell profiles in BALF

**Fig 2:**
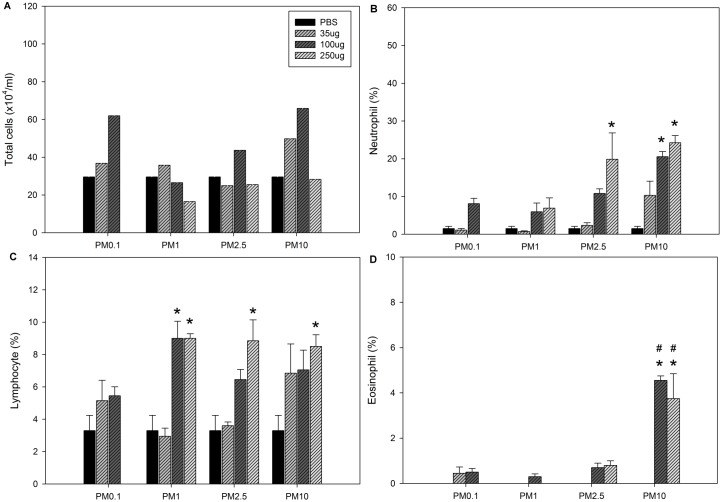
Inflammatory cell profiles in BALF following the 2^nd^ exposure to PM. A: Total cells. B: Neutrophils. C: Lymphocytes. D: Eosinophils. The mice were exposed to 100 μL aliquots of solutions by intratracheal instillation on days 0 and 7 as described in the *Materials and Methods* section. The data are presented as the mean ± SEM (n = 5 per treatment group). Neutrophil, lymphocyte and eosinophil counts are expressed as percentages of total cell counts. *p<0.05 compared to the PBS group; ^#^*p*<0.05 compared to the other groups at the same dose.

BALF inflammatory cell profiles were analyzed three days after the second PM exposure. Neutrophils were significantly increased only in the group exposed to the medium dose of PM_10_. Eosinophils were significantly increased in the medium and high dose PM_10_ exposure groups (Table 3). However, the percentage of neutrophils was significantly increased in the mice treated with a high dose of PM_2.5_ and medium and high doses of PM_10_. The percentage of lymphocytes was significantly increased in the mice treated with a medium dose of PM_1_ and high doses of PM_1_, PM_2.5_, and PM_10_. The percentage of eosinophils was only significantly elevated in the BALF samples of the mice exposed to medium and high doses of PM_10_ (Fig 2).

**Table 3:** Inflammatory cell profiles in BALF following the 2^nd^ exposure to PM.

Treatment	Total cell	Neutrophil	Macrophage	Lymphocyte	Eosinophil
Control	29.6 (±9.2)	0.59 (±0.38)	28.0 (±8.6)	0.95 (±0.33)	0 (±0)
Low dose PM					
PM_0.1_ 35 μg	36.8 (±6.4)	0.32 (±0.06)	34.5 (±6.1)	1.8 (±0.3)	0.14 (±0.09)
PM_1.0_ 35 μg	35.8 (±15.4)	0.23 (±0.09)	34.5 (±14.7)	1.1 (±0.6)	0 (±0)
PM_2.5_ 35 μg	24.9 (±7.4)	0.48 (±0.11)	23.6 (±7.1)	0.9 (±0.2)	0 (±0)
PM_10_ 35 μg	49.8 (±6.1)	4.61 (±1.63)	43.5 (±5.7)	3.5 (±1.4)	0 (±0)
Medium dose PM					
PM_0.1_ 100 μg	62.0 (±12.9)	5.11 (±1.60)	53.0 (±10.8)	3.6 (±0.9)	0.32 (±0.08)
PM_1.0_ 100 μg	26.5 (±2.1)	1.43 (±0.41)	22.6 (±2.0)	2.5 (±0.4)	0.08 (±0.03)
PM_2.5_ 100 μg	43.7 (±10.2)	4.66 (±1.06)	35.6 (±8.5)	2.9 (±0.7)	0.30 (±0.10)
PM_10_ 100 μg	65.9 (±9.0)	13.08 (±1.25)* ^#^	45.2 (±6.8)	4.6 (±1.1)*	3.01 (±0.46)* ^#^
High dose PM					
PM_1.0_ 250 μg	16.6 (±4.4)	1.28 (±0.73)	13.7 (±3.7)	1.5 (±0.4)	0 (±0.0)
PM_2.5_ 250 μg	25.5 (±6.7)	6.83 (±4.28)	16.3 (±2.5)	2.1 (±0.4)	0.19 (±0.06)
PM_10_ 250 μg	28.3 (±6.1)	6.43 (±0.92)	18.2 (±4.3)	2.5 (±0.7)	1.03 (±0.31)* ^#^

The mice were exposed to 100 μL aliquots of solutions by intratracheal instillation on days 0 and 7 as described in the *Materials and Methods* section. The data are presented as the mean ± SEM (10^4^/ml).

*p<0.05 compared to the PBS group

^#^*p*<0.05 compared to the other groups at the same dose (n = 5 for each treatment group)

### Cytokine expression profiles in BALF

**Fig 3:**
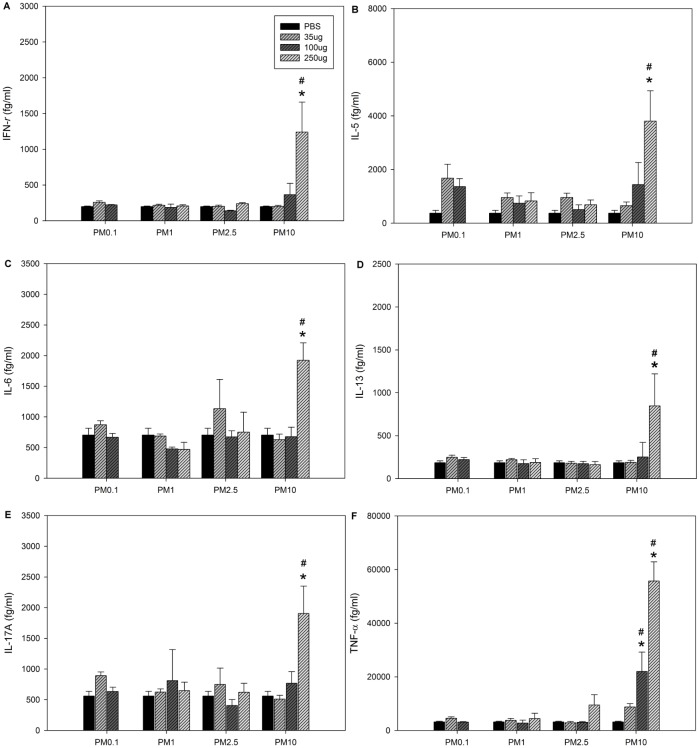
Cytokine levels in BALF following the 2^nd^ exposure to PM. A: IFN-γ. B: IL-5. C: IL-6. D: IL-13. E: IL-17A. F: TNF-α. The mice were exposed to 100 μL aliquots of solutions by intratracheal instillation on days 0 and 7 as described in the *Materials and Methods* section. PBS served as a vehicle control. The data are presented as the mean ± SEM (n = 5 per treatment group). *p<0.05 compared to the PBS control group; ^#^*p*<0.05 compared to the other groups at the same dose.

We next determined the cytokine expression profiles in the BALF samples (Fig 3). We found significantly increased levels of IL-5, IL-13, IL-17A, IL-6, TNF-α and IFN-γ in the high-dose PM_10_ treatment group compared to the control and other treatment groups. TNF-α levels also increased after exposure to a medium dose of PM_10_.

### Histological analysis

**Fig 4:**
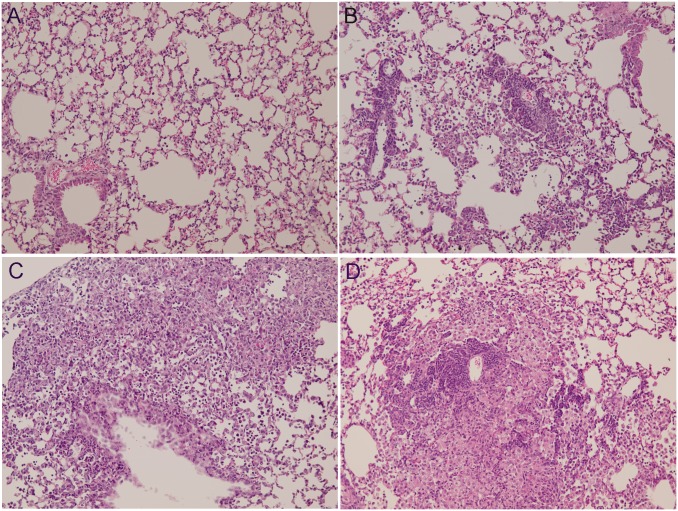
Representative histology of lung tissue (200X) 3 days after the second intratracheal instillation of PM. Lungs were excised and fixed with formaldehyde, sectioned in 5-μm-thick slices, and the slides were stained with H&E. (A) Vehicle (VEH) control tissue. (B) Tissue exposed to high-dose PM_2.5_ showing focal aggregation of lymphocytes and foamy histiocytes in the alveolar space. (C) Tissue exposed to medium-dose PM_10_ showing focal infiltration of lymphocytes and foamy histiocytes into the alveolar space. (D) Tissue exposed to high-dose PM_10_ showing focal infiltration of neutrophils, lymphoplasma cells and foamy histiocytes.

Representative images of H&E-stained lung tissues are shown in Fig 4. Exposure to a high dose of PM_10_ resulted in focal infiltration of neutrophils, lymphoplasma cells and foamy histiocytes. Exposure to a medium dose of PM_10_ resulted in focal infiltration of lymphocytes and foamy histiocytes into the alveolar space. Exposure to a high dose of PM_2.5_ also resulted in focal aggregation of lymphocytes and foamy histiocytes in the alveolar space. The histological changes were minimal in the other exposure groups.

### Associations between PM constituents and inflammatory responses

The coefficients for the correlations between the constituents of the PM samples and the measured inflammatory responses are shown in Table 4. Several ions (Mg^2+^, Ca^2+^, and Cl^-^) had statistically significant positive correlations with elevated percentages of neutrophils, eosinophils and inflammatory cytokines (TNF-α, IFN-γ, and IL-5). Endotoxin levels were also positively associated with elevated percentages of neutrophils and eosinophils as well as augmented TNF-α expression in BALF.

**Table 4:** Coefficients for the correlations between the constituents in the PM samples and the measured inflammatory responses.

Inflammatory biomarker							
	Neutro	Eosino	IFN-γ	TNF-α	IL-5	IL-6	IL-17A
**Na**^**+**^	0.663**	0.809**	0.398**	0.544**	0.309*	0.217	0.331**
**NH**_**4**_^**+**^	0.348**	0.276*	0.143	-0.035	0.026	-0.172	0.099
**K**^**+**^	0.163	0.218	0.181	-0.153	0.198	-0.119	0.178
**Mg**^**2+**^	0.562**	0.73**	0.424**	0.429**	0.427**	0.264*	0.366**
**Ca**^**2+**^	0.54**	0.672**	0.404**	0.467**	0.423**	0.244	0.335**
**Cl**^**-**^	0.569**	0.743**	0.417**	0.448**	0.433**	0.269*	0.372**
**NO**_**3**_^**-**^	0.446**	0.666**	0.314**	0.342**	0.285*	0.224	0.291*
**SO**_**4**_^**2-**^	0.394**	0.309*	0.145	-0.042	0.037	-0.176	0.104
**OC**	0.144	0.313*	0.094	-0.154	0.191	-0.081	0.142
**EC**	0.102	0.259*	0.225	-0.113	0.25	-0.04	0.218
**Endo**	0.796**	0.6**	0.211	0.426**	-0.001	-0.057	0.109

The values are represented as Spearman’s correlation coefficients.

**p<0.01

*p<0.05

Neutro, percentage of neutrophil; Eosino, percentage of eosinophil; Endo, endotoxin.

## Discussion

In this study, we evaluated the effects of PM on the sensitization phase of a murine asthma model. For these experiments, on day 0 and day 7, mice were administered either PBS or different doses of PM_0.1_, PM_1_, PM_2.5_ and PM_10_ samples for allergic sensitization. We found that the percentages of neutrophils in BALF were significantly increased in the mice that were treated with a high dose of PM_2.5_ and medium and high doses of PM_10_. Furthermore, increased levels of TNF-α, IFN-γ, IL-5, IL-13, IL-17A, and IL-6 were found in the BALF samples of the mice that underwent high-dose PM_10_ exposure compared to those of the control and other treatment groups. BALF analysis also showed that exposure to high doses of PM_10_ alone led to elevated numbers and percentages of eosinophils and increased inflammatory cytokine expression (IL-5 and IL-13). Extensive testing of individual PM_2.5_, PM_1_ and PM_0.1_ samples did not reveal an effect on eosinophilic inflammation.

Our results showed that neutrophils and expression levels of inflammatory cytokines (TNF-α, IFN-γ, and IL-6) in BALF were significantly increased in mice treated with high doses of PM_10_ compared with those of the control and other treatment groups. Several epidemiological studies have suggested that coarse PM has a similar or stronger short-term effect on respiratory diseases compared to that of fine PM [29]. A previous study investigating healthy mice submitted to oropharyngeal exposure to PM of different sizes collected from six US cities at doses of 25 μg and 100 μg also showed that coarse PM more potently induced pulmonary inflammation than that of fine or ultrafine PM [30]. Happo et al. [31] also found that coarse PM and PM_2.5–1_ had substantially higher inflammatory potency than PM_1_. Another similar study that compared size-fractioned PM toxicity in samples collected near and far from urban highways showed that coarse PM produced more significant pulmonary inflammation than that of fine and ultrafine PM on a comparative mass basis [25]. Our results (e.g., increased neutrophilia and elevation of IL-6 and TNF-α in BALF) were similar to those reported in the above-cited studies and suggest that PM_10_ exerts a greater inflammatory effect than that of PM_2.5_ on a mass basis. The findings that PM_10_ induced the greatest degree of airway inflammation may be attributed to enriched metal constituents in PM_10_ [32], more biogenic materials with the highest endotoxin contents [31], or chemical contents adhered to PM_10_ [33]. However, many types of combined effects may take place due to complex urban PM.

Our results demonstrated that an allergic immune response, including the recruitment of eosinophils (approximately 4% of total inflammatory cells) and the up-regulation of Th2 cell-related cytokine release, such as IL-5 and IL-13, was observed in the BALF of the mice that were exposed to PM_10_. Although typical models of allergic airway inflammation generally demonstrate much higher percentages of airway eosinophilia, the mice were exposed to PM only in the sensitization phase without further allergen challenge. Additionally, typical allergens (OVA) or adjuvants (aluminum hydroxide) were not used in our experiment. It has previously been shown that a single oropharyngeal exposure to ambient PM (mean hydrodynamic diameter: 0.4 μm) collected from Baltimore city air induced eosinophilic infiltration (<2% of inflammatory cell) in the BALF of mice. In the same study, ambient PM was also shown to activate myeloid dendritic cells (mDCs) and further drive Th2 cytokine responses in naïve T cells [34]. The repeated intratracheal instillation of healthy mice with PM samples from urban Baltimore (aerodynamic diameter <0.85 μm, 0.5 mg in 50 μL PBS) induced airway hyper-responsiveness, small but significant elevations in eosinophil counts in BALF, and increased Th2 expression of IL-17A, IL-5, and IL-13 cytokines in lung cells but no increase in total serum IgE levels [26]. Immature dendritic cells (DCs) are highly effective at taking up foreign bodies and directing the primary immune response [35]. An *in vitro* study showed that exposure to ambient PM enhanced DC activation and that co-cultures of ambient PM-stimulated DCs with alloreactive naïve CD4+ T cells produced Th2-like patterns of cytokine production [36]. A recent in vitro study reported that a co-culture of human myeloid DCs with PM can stimulate autologous human memory CD4 T cells (Tms) to secrete IFN-γ and IL-13 and drive the expansion and differentiation of a mixed population of Th1, Th2 and Th17 effector memory cells [37]. Although the exposure doses, sizes and compositions of the PM samples were different, our observations together with previous studies indicate that exposure to ambient PM can induce allergic airway inflammation.

The inflammatory pathways involved in the development of asthma are complex, and eosinophils play a central role in the pathogenesis of asthma. Eosinophils increase vascular permeability, epithelial damage, mucus hypersecretion, and smooth muscle constriction through their release of granule-associated basic proteins, lipid mediators and reactive oxygen species (ROS) [38]. IL-5 is an important mediator of eosinophil proliferation, differentiation, maturation, migration, and survival in response to environmental stimuli [39]. IL-13 activates macrophages and eosinophils to accelerate inflammation and stimulates B-cells to produce IgE antibodies. IL-13 also contributes to airway hyper-responsiveness and airway remodeling through epithelial cell hypersecretion, subepithelial fibroblast proliferation and smooth muscle hypertrophy in the airway [40]. IL-17A may induce epithelial structural changes and smooth muscle contraction; the presence of IL-17A in BALF has been associated with asthma severity in clinical studies. An association between IL-17A and neutrophilic airway inflammation has also been established in murine asthma models. Collectively, these results suggest that IL-17A plays a central role in the pathophysiologies of certain asthma phenotypes [41].

PMs are composed of carbon cores and a number of soluble and insoluble components, including acids, organic chemicals, metals, endotoxin, pollen or fungi debris on their surface. Thus, the underlying mechanisms by which PM exerts biological effects are complex. Various allergens, such as fungal spores and pollen, are more efficiently carried to the airways when they are constituents of PM [42,43]. PM exposure may impact many cell types at different levels of immune regulation. A Th2-dominant immune response plays a central role in allergic airway inflammation; however, innate immunity, such as that via dendritic cell and epithelial cell responses, was also important in disease expression [44]. PM has been shown to stimulate innate immune responses through airway epithelial cells and dendritic cells [45]. The aryl hydrocarbon receptor (AhR) is a ligand-activated transcription factor that is expressed in a variety of tissues. The activation of the AhR may regulate gene expressions involving detoxication enzymes (CYP1A1 and CYP1B1), as well as inflammatory (TNF-α and IL-6) and T-cell differentiation (Foxp3, IL-17 and GATA3) genes [46]. Polycyclic aromatic hydrocarbons (PAHs) contained in the PMs, common AhR agonists, have been shown to enhance Th17 cell differentiation and IL-17 secretion through the aryl hydrocarbon receptor, indicating that PM can directly modulate T cell function [47]. An *in vitro* study also showed that exposure to ambient PM enhanced DC activation and that co-cultures of ambient PM-stimulated DCs with alloreactive naïve CD4+ T cells produced Th2-like patterns of cytokine production [25,31]. The innate immune cells contain pattern recognition receptors (PRRs), including toll-like receptors (TLRs), nucleotide binding and oligomerization domain (NOD)-like receptors (NLRs), among others [48]. These receptors detect exogenous or endogenous toxic substances by priming and activating the inflammasome, a cytosolic protein complex that can trigger the maturation of pro-inflammatory chemokines and cytokines and assist the host in regulating the balance between tissue repair and inflammation [49]. PM_10_ has been shown to activate an NLRP3 inflammasome/IL-1 receptor axis involving IL-1β, CCL-20 and GM-CSF production, which causes dendritic cell activation and neutrophilia [50]. Additionally, the production of oxidative stress (either the direct induction of reactive oxygen species attributed to organic and metal components of the PM or the secondary induction of local cellular responses after PM exposure) induced the activation of the mitogen-activated protein (MAP) kinase cascade and NF-κB transcription factors, which control the activity of genes involved in cellular activation and inflammation and appear to be fundamental to the PM-induced immunoregulatory effect [51].

As the size of particles decreases, the total surface area increases. Thus, the eosinophilic inflammatory effect of ultrafine particles was reported to be greater than that of fine particles [24]. In our study, PM_10_ induced a more prominent eosinophilic inflammatory response than that of PM_2.5_ or PM_0.1_. Our findings suggest that the compositions of PM may also result in differences. We found that PM_10_ had a higher endotoxin level than that of fine and ultrafine PM and that the endotoxin level was associated with neutrophilic inflammation. Our findings were consistent with those of previous PM studies [25,31]. The underlying mechanism of endotoxin-induced inflammation involves the detection of endotoxin by a complex of CD14, Toll-like receptor 4 (TLR4) and MD-2. The recognition of endotoxin leads to the activation of NF-κB family transcription factors and mitogen-activated protein kinases pathways of innate immune cells, which directs the expression of proinflammatory cytokines, chemokines and adhesion molecules [52,53]. However, the causal relationship between endotoxin and eosinophilic airway inflammation and Th2 immune response are more complex. According to the ‘hygiene hypothesis’, the decreasing incidence of infections is associated with an increasing incidence of allergic disease [54]. However, epidemiological evidence has indicated that endotoxin exposure is a risk factor for increased asthma prevalence [55,56], and animal studies of the immunomodulatory effects of endotoxin have yielded different and sometimes conflicting results, as reviewed by Zhu et al [57]. Several investigators demonstrated that low-dose endotoxin with OVA induced Th2 immune responses, whereas high-dose endotoxin with OVA induced Th1 inflammation [58]. Our current results showed that endotoxin level was positively associated with elevated percentages of neutrophils and eosinophils as well as increased expression of TNF-α in BALF. It is possible that the dose, route and timing of endotoxin exposure may determine subsequent inflammatory responses.

In the current study, we found that select PM constituents showed statistical correlations with inflammatory responses. However, PM constituents from the same sources are also complex, and they may interact with each other. Therefore, it is difficult to determine the causal relationship between any single component that is significantly associated with inflammation; as such, investigations into the relationships that exist between particle sources and health effects should be carried out [59].

Taken together, our results indicate that ambient PM can induce Th1/Th2/Th17 inflammatory responses through different additive or synergistic mechanisms driven both by individual compounds and combinations of compounds.

## Conclusions

Due to wide variations in the sources and compositions of PM as well as the different PM exposure protocols used in the previous animal studies, there is a lack of consistent findings regarding the physiological effects of PM in the airway. In the current study, we found that exposure to PM increased inflammatory responses, including augmented neutrophil infiltration and increased TNF-α and IFN-γ production, as well as allergic immune responses, including augmented eosinophil infiltration and increased production of Th2-related cytokines (IL-5 and IL-13), in the BALF of mice that were repeatedly exposed to PM_10_ in the absence of an additional OVA. Our results further indicate that ambient PM exposure may increase asthma morbidity.

## Supporting information

S1 File Inflammatory cell profiles in BALF. (XLS)

S2 File Cytokine levels in BALF. (XLS)

## References

[1] U.S. EPA. Air quality criteria for particulate matter. Washington, DC: U.S. Environmental Protection Agency; 2004.

[2] Kelly FJ, Fussell JC. Size, source and chemical composition as determinants of toxicity attributable to ambient particulate matter. Atmos Environ. 2012;60: 504–526.

[3] Riedl M, Diaz-Sanchez D. Biology of diesel exhaust effects on respiratory function. J Allergy Clin Immunol. 2005;115: 221–228; quiz 229. 15696072 10.1016/j.jaci.2004.11.047

[4] Brook RD, Rajagopalan S, Pope CA, Brook JR, Bhatnagar A, Diez-Roux AV, et al Particulate matter air pollution and cardiovascular disease: an update to the scientific statement from the American Heart Association. Circulation. 2010;121: 2331–2378. 10.1161/CIR.0b013e3181dbece1 20458016

[5] Pope CA, Burnett RT, Thun MJ, Calle EE, Krewski D, Ito K, et al Lung cancer, cardiopulmonary mortality, and long-term exposure to fine particulate air pollution. JAMA. 2002;287: 1132–1141. 11879110 10.1001/jama.287.9.1132 PMC4037163

[6] Ling SH, van Eeden SF. Particulate matter air pollution exposure: role in the development and exacerbation of chronic obstructive pulmonary disease. Int J Chron Obstruct Pulmon Dis. 2009;4: 233–243. 19554194 10.2147/copd.s5098 PMC2699820

[7] Bateman ED, Hurd SS, Barnes PJ, Bousquet J, Drazen JM, FitzGerald M, et al Global strategy for asthma management and prevention: GINA executive summary. Eur Respir J. 2008;31: 143–178. 10.1183/09031936.00138707 18166595

[8] Bousquet J, Bousquet PJ, Godard P, Daures JP. The public health implications of asthma. Bull World Health Org. 2005;83: 548–554. 16175830 PMC2626301

[9] Lee YL, Hwang BF, Lin YC, Guo YL, Taiwan Childhood Allergy Survey Group. Time trend of asthma prevalence among school children in Taiwan [corrected]. Pediatr Allergy Immunol. 2007;18: 188–195. 10.1111/j.1399-3038.2006.00504.x 17432997

[10] Asher MI, Montefort S, Bjorksten B, Lai CK, Strachan DP, Weiland SK, et al Worldwide time trends in the prevalence of symptoms of asthma, allergic rhinoconjunctivitis, and eczema in childhood: ISAAC Phases One and Three repeat multicountry cross-sectional surveys. Lancet. 2006;368: 733–743. 16935684 10.1016/S0140-6736(06)69283-0

[11] Goldsmith CA, Kobzik L. Particulate air pollution and asthma: a review of epidemiological and biological studies. Rev Environ Health. 1999;14: 121–134. 10674285 10.1515/reveh.1999.14.3.121

[12] Lin M, Chen Y, Burnett RT, Villeneuve PJ, Krewski D. The influence of ambient coarse particulate matter on asthma hospitalization in children: case-crossover and time-series analyses. Environ Health Perspect. 2002;110: 575–581. 12055048 10.1289/ehp.02110575 PMC1240873

[13] Peel JL, Tolbert PE, Klein M, Metzger KB, Flanders WD, Todd K, et al Ambient air pollution and respiratory emergency department visits. Epidemiology. 2005;16: 164–174. 15703530 10.1097/01.ede.0000152905.42113.db

[14] Gauderman WJ, Avol E, Gilliland F, Vora H, Thomas D, Berhane K, et al The effect of air pollution on lung development from 10 to 18 years of age. N Engl J Med. 2004;351: 1057–1067. 10.1056/NEJMoa040610 15356303

[15] Clark NA, Demers PA, Karr CJ, Koehoorn M, Lencar C, Tamburic L, et al Effect of early life exposure to air pollution on development of childhood asthma. Environ Health Perspect. 2010;118: 284–290. 10.1289/ehp.0900916 20123607 PMC2831931

[16] Heinrich J, Hoelscher B, Wjst M, Ritz B, Cyrys J, Wichmann H. Respiratory diseases and allergies in two polluted areas in East Germany. Environ Health Perspect. 1999;107: 53–62. 9872717 10.1289/ehp.9910753 PMC1566314

[17] Dong GH, Chen T, Liu MM, Wang D, Ma YN, Ren WH, et al Gender differences and effect of air pollution on asthma in children with and without allergic predisposition: northeast Chinese children health study. PLoS One. 2011;6: e22470 10.1371/journal.pone.0022470 21811617 PMC3139656

[18] Landrigan PJ, Lioy PJ, Thurston G, Berkowitz G, Chen LC, Chillrud SN, et al Health and environmental consequences of the world trade center disaster. Environ Health Perspect. 2004;112: 731–739. 15121517 10.1289/ehp.6702 PMC1241968

[19] Alexis NE, Lay JC, Zeman K, Bennett WE, Peden DB, Soukup JM, et al Biological material on inhaled coarse fraction particulate matter activates airway phagocytes in vivo in healthy volunteers. J Allergy Clin Immunol. 2006;117: 1396–1403. 16751003 10.1016/j.jaci.2006.02.030

[20] Maes T, Provoost S, Lanckacker EA, Cataldo DD, Vanoirbeek JA, Nemery B, et al Mouse models to unravel the role of inhaled pollutants on allergic sensitization and airway inflammation. Respir Res. 2010;11: 7 10.1186/1465-9921-11-7 20092634 PMC2831838

[21] Gavett SH, Haykal-Coates N, Copeland LB, Heinrich J, Gilmour MI. Metal composition of ambient PM2.5 influences severity of allergic airways disease in mice. Environ Health Perspect. 2003;111: 1471–1477. 12948886 10.1289/ehp.6300 PMC1241649

[22] Li N, Harkema JR, Lewandowski RP, Wang M, Bramble LA, Gookin GR, et al Ambient ultrafine particles provide a strong adjuvant effect in the secondary immune response: implication for traffic-related asthma flares. Am J Physiol Lung Cell Mol Physiol. 2010;299: L374–383. 10.1152/ajplung.00115.2010 20562226 PMC2951067

[23] Steerenberg PA, Withagen CE, van Dalen WJ, Dormans JA, Heisterkamp SH, van Loveren H, et al Dose dependency of adjuvant activity of particulate matter from five European sites in three seasons in an ovalbumin-mouse model. Inhal Toxicol. 2005;17: 133–145. 10.1080/08958370590904490 15788374

[24] Li N, Wang M, Bramble LA, Schmitz DA, Schauer JJ, Sioutas C, et al The adjuvant effect of ambient particulate matter is closely reflected by the particulate oxidant potential. Environ Health Perspect. 2009;117: 1116–1123. 10.1289/ehp.0800319 19654922 PMC2717139

[25] Cho SH, Tong H, McGee JK, Baldauf RW, Krantz QT, Gilmour MI. Comparative toxicity of size-fractionated airborne particulate matter collected at different distances from an urban highway. Environ Health Perspect. 2009;117: 1682–1689. 10.1289/ehp.0900730 20049117 PMC2801189

[26] Saunders V, Breysse P, Clark J, Sproles A, Davila M, Wills-Karp M. Particulate matter-induced airway hyperresponsiveness is lymphocyte dependent. Environ Health Perspect. 2010;118: 640–646. 10.1289/ehp.0901461 20061214 PMC2866679

[27] Huang KL, Lee YH, Chen HI, Liao HS, Chiang BL, Cheng TJ. Zinc oxide nanoparticles induce eosinophilic airway inflammation in mice. J Hazard Mater. 2015;297: 304–312. 26010476 10.1016/j.jhazmat.2015.05.023

[28] Gugamsetty B, Wei H, Liu C-N, Awasthi A, Hsu S-C, Tsai C-J, et al Source characterization and apportionment of PM10, PM2.5 and PM0.1 by using positive matrix factorization. Aerosol Air Qual Res. 2012;12: 476–491.

[29] Brunekreef B, Forsberg B. Epidemiological evidence of effects of coarse airborne particles on health. Eur Respir J. 2005;26: 309–318. 10.1183/09031936.05.00001805 16055881

[30] Gilmour MI, McGee J, Duvall RM, Dailey L, Daniels M, Boykin E, et al Comparative toxicity of size-fractionated airborne particulate matter obtained from different cities in the United States. Inhal Toxicol. 2007;19 Suppl 1: 7–16. 17886044 10.1080/08958370701490379

[31] Happo MS, Hirvonen MR, Halinen AI, Jalava PI, Pennanen AS, Sillanpaa M, et al Seasonal variation in chemical composition of size-segregated urban air particles and the inflammatory activity in the mouse lung. Inhal Toxicol. 2010;22: 17–32. 10.3109/08958370902862426 20017591

[32] Dye JA, Lehmann JR, McGee JK, Winsett DW, Ledbetter AD, Everitt JI, et al Acute pulmonary toxicity of particulate matter filter extracts in rats: coherence with epidemiologic studies in Utah Valley residents. Environ Health Perspect. 2001;109 Suppl 3: 395–403. 11427389 10.1289/ehp.01109s3395 PMC1240557

[33] Schlesinger RB, Cassee F. Atmospheric secondary inorganic particulate matter: the toxicological perspective as a basis for health effects risk assessment. Inhal Toxicol. 2003;15: 197–235. 10.1080/08958370304503 12579454

[34] Bezemer GF, Bauer SM, Oberdorster G, Breysse PN, Pieters RH, Georas SN, et al Activation of pulmonary dendritic cells and Th2-type inflammatory responses on instillation of engineered, environmental diesel emission source or ambient air pollutant particles in vivo. J Innate Immun. 2011;3: 150–166. 10.1159/000321725 21099199 PMC3072202

[35] Vermaelen K, Pauwels R. Pulmonary dendritic cells. Am J Respir Crit Care Med. 2005;172: 530–551. 10.1164/rccm.200410-1384SO 15879415

[36] Williams MA, Porter M, Horton M, Guo J, Roman J, Williams D, et al Ambient particulate matter directs nonclassic dendritic cell activation and a mixed TH1/TH2-like cytokine response by naive CD4+ T cells. J Allergy Clin Immunol. 2007;119: 488–497. 17187851 10.1016/j.jaci.2006.10.022

[37] Matthews NC, Pfeffer PE, Mann EH, Kelly FJ, Corrigan CJ, Hawrylowicz CM, et al Urban particulate matter-activated human dendritic cells induce the expansion of potent inflammatory Th1, Th2, and Th17 effector cells. Am J Respir Cell Mol Biol. 2016;54: 250–262. 10.1165/rcmb.2015-0084OC 26196219 PMC4821044

[38] Park YM, Bochner BS. Eosinophil survival and apoptosis in health and disease. Allergy Asthma Immunol Res. 2010;2: 87–101. 10.4168/aair.2010.2.2.87 20358022 PMC2846745

[39] Rosenberg HF, Phipps S, Foster PS. Eosinophil trafficking in allergy and asthma. J Allergy Clin Immunol. 2007;119: 1303–1310; quiz 1311–1302. 17481712 10.1016/j.jaci.2007.03.048

[40] Ingram JL, Kraft M. IL-13 in asthma and allergic disease: asthma phenotypes and targeted therapies. J Allergy Clin Immunol. 2012;130: 829–842; quiz 843–824. 22951057 10.1016/j.jaci.2012.06.034

[41] Chesne J, Braza F, Mahay G, Brouard S, Aronica M, Magnan A. IL-17 in severe asthma. Where do we stand? Am J Respir Crit Care Med. 2014;190: 1094–1101. 10.1164/rccm.201405-0859PP 25162311

[42] Delfino RJ, Zeiger RS, Seltzer JM, Street DH, Matteucci RM, Anderson PR, et al The effect of outdoor fungal spore concentrations on daily asthma severity. Environ Health Perspect. 1997;105: 622–635. 9288497 10.1289/ehp.97105622 PMC1470068

[43] Ostro B, Lipsett M, Mann J, Braxton-Owens H, White M. Air pollution and exacerbation of asthma in African-American children in Los Angeles. Epidemiology. 2001;12: 200–208. 11246581 10.1097/00001648-200103000-00012

[44] Lambrecht BN, Hammad H. The role of dendritic and epithelial cells as master regulators of allergic airway inflammation. Lancet. 2010;376: 835–843. 20816550 10.1016/S0140-6736(10)61226-3

[45] de Haar C, Kool M, Hassing I, Bol M, Lambrecht BN, Pieters R. Lung dendritic cells are stimulated by ultrafine particles and play a key role in particle adjuvant activity. J Allergy Clin Immunol. 2008;121: 1246–1254. 18313130 10.1016/j.jaci.2008.01.010

[46] Hao N, Whitelaw ML. The emerging roles of AhR in physiology and immunity. Biochem Pharmacol. 2013;86: 561–570. 23856287 10.1016/j.bcp.2013.07.004

[47] van Voorhis M, Knopp S, Julliard W, Fechner JH, Zhang X, Schauer JJ, et al Exposure to atmospheric particulate matter enhances Th17 polarization through the aryl hydrocarbon receptor. PLoS One. 2013;8: e82545 10.1371/journal.pone.0082545 24349309 PMC3859609

[48] Barbe F, Douglas T, Saleh M. Advances in Nod-like receptors (NLR) biology. Cytokine Growth Factor Rev. 2014;25: 681–697. 25070125 10.1016/j.cytogfr.2014.07.001

[49] Lamkanfi M, Dixit VM. Mechanisms and functions of inflammasomes. Cell. 2014;157: 1013–1022. 24855941 10.1016/j.cell.2014.04.007

[50] Hirota JA, Gold MJ, Hiebert PR, Parkinson LG, Wee T, Smith D, et al The nucleotide-binding domain, leucine-rich repeat protein 3 inflammasome/IL-1 receptor I axis mediates innate, but not adaptive, immune responses after exposure to particulate matter under 10 mum. Am J Respir Cell Mol Biol. 2015;52: 96–105. 10.1165/rcmb.2014-0158OC 24988285

[51] Ghio AJ, Carraway MS, Madden MC. Composition of air pollution particles and oxidative stress in cells, tissues, and living systems. J Toxicol Environ Health B Crit Rev. 2012;15: 1–21. 10.1080/10937404.2012.632359 22202227

[52] Strieter RM, Belperio JA, Keane MP. Cytokines in innate host defense in the lung. J Clin Invest. 2002;109: 699–705. 10.1172/JCI15277 11901175 PMC150916

[53] Arthur JS, Ley SC. Mitogen-activated protein kinases in innate immunity. Nat Rev Immunol. 2013;13: 679–692. 10.1038/nri3495 23954936

[54] Strachan DP. Hay fever, hygiene, and household size. BMJ. 1989;299: 1259–1260. 2513902 10.1136/bmj.299.6710.1259 PMC1838109

[55] Thorne PS, Kulhankova K, Yin M, Cohn R, Arbes SJ Jr., Zeldin DC. Endotoxin exposure is a risk factor for asthma: the national survey of endotoxin in United States housing. Am J Respir Crit Care Med. 2005;172: 1371–1377. 10.1164/rccm.200505-758OC 16141442 PMC1379232

[56] Celedon JC, Milton DK, Ramsey CD, Litonjua AA, Ryan L, Platts-Mills TA, et al Exposure to dust mite allergen and endotoxin in early life and asthma and atopy in childhood. J Allergy Clin Immunol. 2007;120: 144–149. 17507083 10.1016/j.jaci.2007.03.037 PMC3737770

[57] Zhu Z, Oh SY, Zheng T, Kim YK. Immunomodulating effects of endotoxin in mouse models of allergic asthma. Clin Exp Allergy. 2010;40: 536–546. 10.1111/j.1365-2222.2010.03477.x 20447074

[58] Kim YK, Oh SY, Jeon SG, Park HW, Lee SY, Chun EY, et al Airway exposure levels of lipopolysaccharide determine type 1 versus type 2 experimental asthma. J Immunol. 2007;178: 5375–5382. 17404323 10.4049/jimmunol.178.8.5375

[59] Happo MS, Hirvonen MR, Halinen AI, Jalava PI, Pennanen AS, Sillanpaa M, et al Chemical compositions responsible for inflammation and tissue damage in the mouse lung by coarse and fine particulate samples from contrasting air pollution in Europe. Inhal Toxicol. 2008;20: 1215–1231. 10.1080/08958370802147282 18855153

